# A low-cost, easy-to-implement optical add-on to convert a retinal OCT into a ultra-high-resolution corneal OCT

**DOI:** 10.1016/j.zemedi.2025.09.004

**Published:** 2025-10-10

**Authors:** Fritz Stange, Sebastian Bohn, Alois Gottschlich, Tobias Korn, Oliver Stachs, Karsten Sperlich

**Affiliations:** a Department of Ophthalmology, Rostock University Medical Center, 18057 Rostock, Germany; b Institute of Physics, University of Rostock 18059 Rostock, Germany; c Department Life, Light & Matter, University of Rostock 18059 Rostock, Germany

**Keywords:** Cornea, anterior segment, OCT, high-resolution, EBMD

## Abstract

**Purpose:**

Commercial anterior segment optical coherence tomography (OCT) systems typically offer sufficient axial resolution to distinguish corneal layers. However, their lateral resolution (∼15–20 µm) is limited, restricting the visualization of fine microstructural features, relevant for noninvasive diagnosis of corneal pathologies. We present a low-cost, easy-to-implement optical add-on that substantially improves the lateral resolution of a standard retinal OCT system, enabling high-fidelity corneal imaging.

**Methods:**

Custom-designed optical modules were developed for the standard objective lens of an investigational retinal OCT device. The system design, incorporating a single achromatic lens, was optimized through optical simulations to achieve a higher numerical aperture and reduced focal length. The lateral resolution was evaluated both theoretically, via point spread function, and experimentally using a 1951 United States Air Force (USAF) resolution target. A compact prototype, presented here, was tested on a commercial SPECTRALIS OCT2, compared with the commercial Anterior Segment Module (both Heidelberg Engineering GmbH, Heidelberg, Germany), and tested in vivo on two human subjects, including one with suspected epithelial basement membrane dystrophy (EBMD).

**Results:**

The prototype achieved a lateral resolution of 3.1 µm in the experiment, closely matching the results of the optical simulation. This resolution is markedly better than the 15.6 µm achieved with the commercial corneal OCT, albeit at a cost of a reduced field of view (2.62 mm vs. 8.29 mm). In vivo imaging confirmed enhanced visualization of epithelial microstructures in both healthy and pathological corneas, despite a reduced depth of focus resulting from the higher numerical aperture.

**Conclusion:**

The proposed add-on enabled ultra-high-resolution corneal imaging on a standard SPECTRALIS OCT2 platform without the need for major hardware modifications, such as an increased reference arm or dispersion compensation. This low-cost, easy-to-implement approach offers diagnostic value for early detection and longitudinal monitoring of subtle corneal irregularities, particularly in conditions such as EBMD, where a high lateral resolution is critical for clinical decision-making.

## Introduction

Optical coherence tomography (OCT), originally developed for retinal imaging [1], has also become a cornerstone of modern anterior segment diagnostics of the eye. For anterior segment applications, the objective lens typically need to be replaced, and the reference arm adjusted to account for the different optical path length. These adaptations allow OCT to provide ultra-high-resolution, cross-sectional imaging of corneal structures in a non-invasive, real-time manner. In clinical anterior segment OCT, the axial resolution of the Anterior Segment Module (ASM, Heidelberg Engineering GmbH, Heidelberg, Germany) attached to the SPECTRALIS OCT platform (Heidelberg Engineering GmbH, Heidelberg, Germany) typically ranges from 3.9–7 µm [2], in corneal tissue, achieving a lateral resolution of 14 µm [2]. Other commercial devices range from about 5 µm to 10 µm axial and 15 µm to <45 µm lateral resolution [3]. Such values suffice for coarse layer discrimination but not for resolving fine epithelial or stromal microstructures.

In clinical practice, several corneal conditions require enhanced lateral detail for precise assessment and disease management. For instance, in corneal ulcers with imminent perforation, accurate measurement of residual stromal thickness and localization of Descemetoceles is crucial for timely surgical intervention [4]. Similarly, corneal neovascularization, common in contact lens intolerance, post-inflammatory states, or graft rejection, requires precise delineation of vessel depth and perfusion patterns to guide fine-needle diathermy (FND) or anti-VEGF therapy [5,6].

Other scenarios, such as epithelial basement membrane dystrophy (EBMD) [7], band keratopathy [8], Salzmann nodular degeneration [9], and embedded corneal foreign bodies [10] also benefit from ultra-high-resolution imaging, particularly when evaluating lesion depth, epithelial disruption, or stromal involvement [[7], [8], [9], [10], [11]]. EBMD, for instance, often presents with irregular, thickened epithelial profiles that can be mapped in detail using ultra-high-resolution OCT [7,12,13], while Salzmann nodules appear as hyperreflective, subepithelial fibrotic lesions with overlying epithelial thinning [9].

Ultra-high-resolution OCT systems [14,15] have demonstrated the ability to visualize fine epithelial features. However, such systems are often cost-prohibitive and technically complex, limiting their use in routine clinical settings. To address these limitations, we present a low-cost and easy-to-implement optical add-on for a conventional anterior segment OCT device (SPECTRALIS OCT, Heidelberg Engineering GmbH, Heidelberg, Germany) to obtain ultra-high-resolution corneal images, without major hardware modifications. By integrating a custom-designed optical module with an increased numerical aperture and a reduced field of view (FOV), our setup achieves lateral resolutions in the range of 2–3 µm, sufficient to visualize microstructural details that are otherwise inaccessible to standard systems. This approach preserves compatibility with existing clinical workflows while providing focused, high-fidelity corneal imaging.

This study describes the design and implementation of the optical add-on and evaluates its potential clinical utility, using EBMD as one example for a condition where subtle epithelial irregularities are clinically relevant. Previous studies have shown that ultra-high-resolution OCT can detect typical epithelial thickness abnormalities in EBMD and may assist in identifying patients at risk of suboptimal refractive outcomes - particularly in the context of LASIK [7,16].

## Material and methods

The concept involves modifying a retinal OCT system with an optical add-on to shift the imaging focus from the retina to the cornea. This adjustment reduces the effective focal length, thereby increasing the lateral magnification of the corneal image. Our study is based on the SPECTRALIS OCT platform (Heidelberg Engineering GmbH, Heidelberg, Germany). It combines confocal scanning laser ophthalmoscopy (cSLO) and spectral domain OCT utilizing a superluminescent diode (SLD) as a broadband light source. We used an investigational research device named SPECTRALIS HighRes OCT. It features a spectrally broader SLD (839 nm center wavelength and 141 nm bandwidth, measured with AVASPEC-ULS4096CL-EVO, Avantes, Apeldoorn, The Netherlands), improving the axial resolution and an extended reference arm offering more flexibility in terms of optical path length (OPL) of the optical add-on design. The axial resolution in the cornea was calculated to be 3.2 µm, based on its refractive index (n=1.376, [17]) and the spectral power distribution. We also used a commercial SPECTRALIS OCT2 to evaluate the prototype’s compatibility with available devices. The results obtained with the prototype are compared to the commercial Anterior Segment Module (ASM, Heidelberg Engineering GmbH, Heidelberg, Germany).

To obtain a low-cost add-on, the corneal imaging prototype is based on an objective lens for retinal imaging [HE], which emits a collimated beam when set at 0 dpt. The optical add-on is used to focus this beam onto the cornea to achieve a telecentric ultra-high-resolution corneal imaging system.

In OCT, the axial resolution is limited by the spectral bandwidth of the light source and is decoupled from the lateral resolution [18]. The lateral resolution δx is determined by the spot size of the sample beam, which is approximated as a Gaussian beam. The spot size is defined as the radius $\omega_{0}$ of the beam waist at which the intensity has decreased to 1/e^2^ of its peak value. Using the focal length of the optical system *f*_sys_ and the beam diameter *d*, an expression can be derived that depends mainly on the numerical aperture *NA* = *n* · sin(*θ*) of the optical system [19]:

$$\delta x=\sqrt{2\ln2}\omega_{0}=\sqrt{2\ln2}\frac{2\lambda_{0}}{\pi \;}\frac{f_{\text{sys}}}{n\cdot d}=\sqrt{2\ln2}\frac{\lambda_{0}}{\pi \;\cdot NA}$$

According to the equation above and using the same wavelength, an improved lateral resolution requires an increased *NA*. The *NA* is defined by the ratio of the beam diameter, which is fixed, and the focal length. Therefore, in a low-cost approach, the lateral resolution can only be improved by a reduction of the focal length.

Different optical designs based on the standard objective lens and the High Magnification Module (HMM) were simulated in Ansys Zemax OpticStudio (Version 23.2.1, Ansys Inc., Canonsburg, PA, USA), and the point spread function (PSF) as a measure of image quality was calculated. Using the PSF, a theoretical lateral resolution is given by the FWHM [20].

The optimal distance between the base lens and the additional lens combination, and the working distance can be determined using ray tracing. The optimal distance is found when the focus on the image plane is best. The working distance is then given by the distance between the last surface of the additional lens and the image plane. Most designs require an extended reference arm, rendering these prototypes incompatible with any commercial SPECTRALIS devices. Further, the additional amount of glass required a dispersion compensation in most of our experimental realizations to obtain a high image quality.

Here, we present the prototype that, due to its compact design, is compatible with commercial SPECTRALIS systems.

**Fig. 1:**
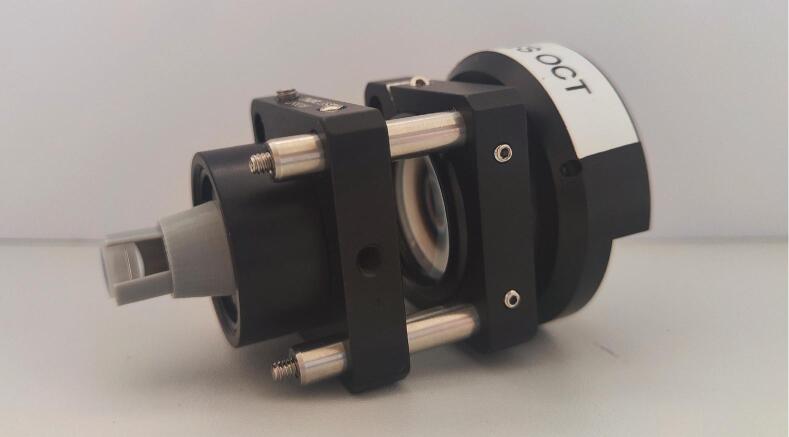
Photograph of the prototypic optical add-on mounted on the standard objective lens for ultra-high-resolution corneal OCT.

It consists of the standard SPECTRALIS objective lens for retinal imaging and a single additional achromatic lens attached in front of it (cf. Fig. 1). The focal length of the standard objective lens was determined to be about 30 mm, and the collimated beam diameter about 3 mm. These values were used for the optical simulation. The additional achromatic lens (AC080-010-B, Thorlabs Inc., Newton, NJ, USA) has a focal length of 10 mm, 8 mm diameter, and a suitable anti-reflective coating ranging from 650 nm to 1050 nm to suppress disruptive back reflections. The lens is oriented such that its more curved surface faces the collimated beam. To mount the lens on the standard objective, a cage plate (CP35, Thorlabs Inc., Newton, NJ, USA) was modified so that it can be clamped on the objective. With four cage system rods (ER1.5, Thorlabs Inc., Newton, NJ, USA), a second cage plate (CP35, Thorlabs Inc., Newton, NJ, USA) holds a lens tube (SM05L05, Thorlabs Inc., Newton, NJ, USA) equipped with a custom 3D-printed holder (base diameter 12.7 mm, length 25 mm). This allows the lens to be quickly mounted onto or removed from the standard objective, enables quick removal of the entire assembly, and enables axial adjustment of the optical add-on relative to the objective lens for optimal positioning. Correct adjustment of the axial distance is important to obtain a planar and undistorted image. It should be noted that the reference arm must be adjusted to the increased optical path length of the prototype compared to the standard objective lens. In the case of the SPECTRALIS platform, this is easily performed by pressing the combination “Ctrl + Alt + Shift + O” and adjusting the position of the reference mirror [21].

The lateral resolution achieved was determined using a 1951 United States Air Force Resolution Test Chart (USAF resolution target). The known linewidth of the individual elements of the USAF resolution target allowed for the determination of the lateral scale (µm/pixel) and the corresponding FOV. To determine the lateral optical resolution, an OCT B-scan line over a group of elements of the USAF resolution target was used. Each element consists of three lines, so three peaks must be visible in the intensity plot of an element's intensity distribution to be considered resolved. The experimental resolution limit was defined as the smallest element that could be resolved.

Subsequently, the prototype was evaluated on the human eye. The first subject, a 41-year-old healthy male, was examined using both the prototype and the ASM. The second subject, a 59-year-old male diagnosed with a presumed early stage of EBMD [12], was examined using the prototype only.

## Results

For the prototype presented here, and using the input parameters described above, the optical simulation yielded in a distance of 30. mm between the objective lens and the base lens. This distance, together with the optical path length added by the achromatic lens (9.78 mm), is within the range that the adjustable reference arm can compensate. Additionally, the working distance was determined to be 6.9 mm, which is sufficient for safe non-contact operation.

Initial recordings with other prototypes exhibited a low image quality, which was impaired by dispersion effects. This was especially the case for variants using two achromatic doublets in the optical add-on. These effects could be corrected by providing modified dispersion correction files to the software.

Using only a single achromatic or aspheric lens instead, as presented here, the influence of the dispersion on image quality was negligible, and no correction had to be taken into account.

**Fig. 2:**
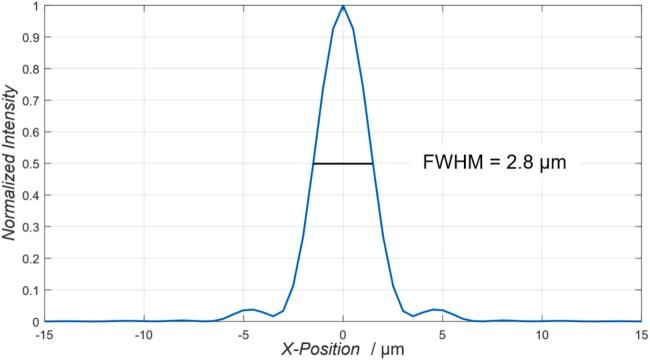
Simulated point spread function of the prototype. The FWHM of the PSF determines the theoretical resolution of the objective lens.

The theoretical lateral resolution was determined from the FWHM of the simulated PSF to be 2.8 µm (cf. Fig. 2). The numerical aperture of the system is 0.15.

**Fig. 3:**
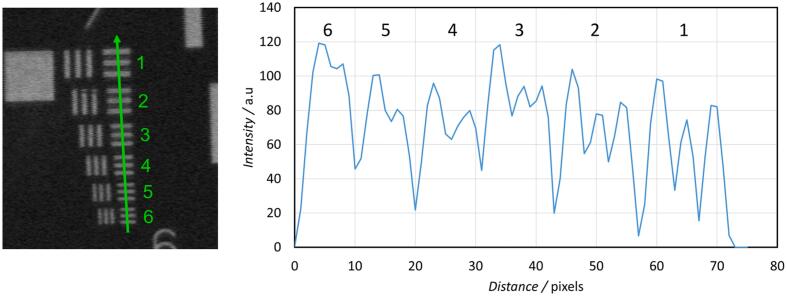
Determination of optical OCT resolution: cSLO image of the USAF resolution target (group 7 with the elements 1 to 6) and OCT b-scan line (left) and OCT intensity distribution along the b-scan line over group 7 (right). The elements 1 to 3 show 3 maxima and are considered resolved. The elements 4 to 6 cannot be resolved.

The experimental determination of the lateral OCT resolution using the USAF resolution target for the prototype is presented in Fig. 3. The USAF resolution target and the OCT b-scan line over the elements of group 7 are shown on the left, while the OCT intensity along this line is presented on the right. Six distinct groups of maxima are observed, corresponding to the respective elements. To be considered resolved, an element must exhibit three discrete maxima. Element 3 contains the smallest resolvable features and determines the resolution limit of the prototype corresponding to the line width of the element. The lateral resolution was determined this way to be 3.1 µm, and the field of view was 2.62 mm. The relative measurement uncertainty of the resolution limit was estimated to be 12%, which corresponds to the relative size increment between adjacent elements in the USAF resolution target.

These parameters are compared with those obtained for the commercial ASM in Table 1. Deviations between the theoretical and experimental resolution are most likely due to imprecise optical simulation input parameters and the experimental step-wise determination of the resolution using the USAF resolution target.

**Table 1:** Comparison between the presented prototype and the commercial ASM.

Objective lens	Lateral resolution limit		USAF Element (group.element)	FOV
	Theoretical	Experimental		
Prototype	2.8 µm	3.1 ± 0.4 µm	7.3	2.62 mm
ASM	-	15.6 ± 1.9 µm	5.1	8.29 mm

**Fig. 4:**
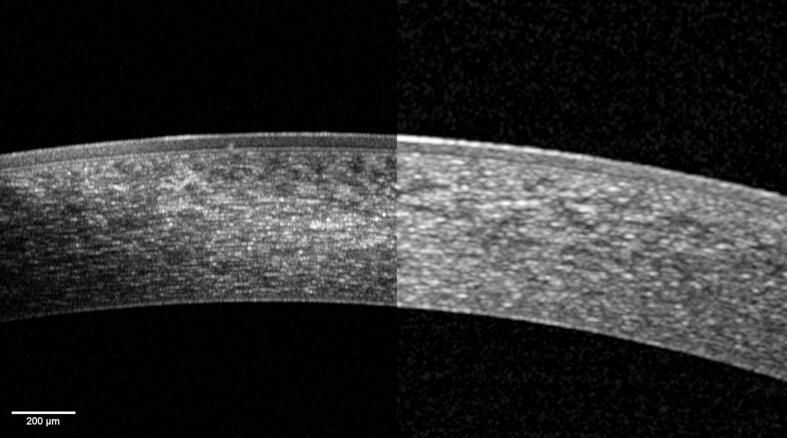
Comparison of the different resolutions achieved using the prototype (left; 3.1 µm) and ASM (right; 15.6 µm), demonstrated with a commercial SPECTRALIS OCT2. Both images were taken from the left cornea of a healthy 41-year-old volunteer. Please note that the actual FOV of both images was truncated, and the images are scaled to the same scale for better visualization of the image quality. Further, the basal epithelium is represented as a flat dark layer.

The improved lateral resolution is demonstrated in Fig. 4, showing a corneal OCT image of the right cornea of subject 1 (healthy, 41 years old) taken with the prototype (left) and the commercial ASM (right) at the same scale. To verify the compatibility with commercial SPECTRALIS platforms, the underlying images were captured with a SPECTRALIS OCT2.

**Fig. 5:**
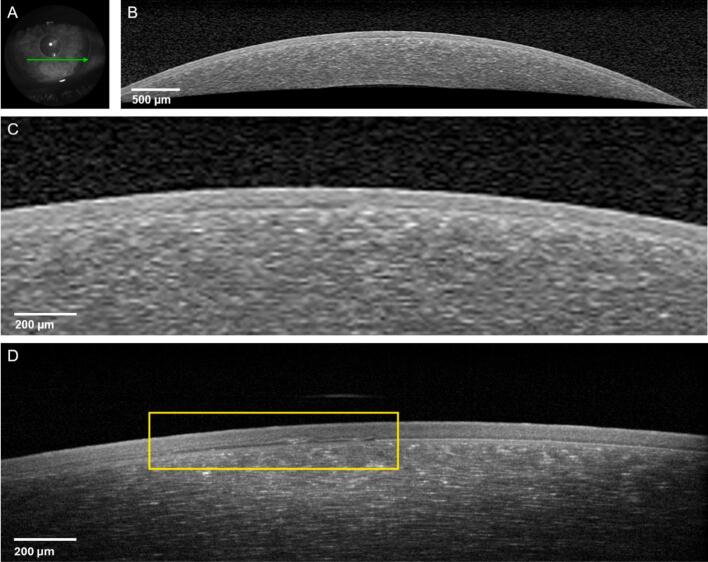
**A and B show i**mages of the right eye of the 59-year-old volunteer captured with the ASM attached to the SPECTRALIS HighRes OCT. A shows the cSLO image of the cornea and the b-scan line in yellow, B presents the corresponding OCT image with the full FOV, and C shows an enlarged region of the cornea. D shows an OCT image of the same eye as above, taken with the SPECTRALIS HighRes OCT and the prototype optical add-on instead of the ASM. The yellow-framed region highlights structural irregularities between epithelium and stroma, indicating pathological findings, presumably EBMD.

Furthermore, the right cornea of a 59-year-old subject with suspected epithelial EBMD was examined using the SPECTRALIS HighRes OCT (see Fig. 5). Using the ASM, the OCT b-scan line is shown in the cSLO image (Fig. 5 A) and the corresponding OCT image is presented in Fig.5 B, demonstrating its FOV. The OCT images exhibit a dark zone between the epithelium and the stroma. However, even in the magnified image (Fig. 5 C), the structure appears blurred, making it difficult to distinct between pathological or physiological features - especially knowing that the basal epithelium is represented as a dark layer (cf. Fig. 4).

Fig. 5 D presents the same cornea imaged with the SPECTRALIS HighRes OCT but with the prototype optical add-on instead of the ASM. The dark region displays structural irregularities (highlighted in the yellow frame) that are likely associated with EBMD. Only the higher lateral resolution enables imaging these fine structural changes in the epithelial structure. Due to the volunteer’s refusal of genetic testing, the suspected diagnosis could not be confirmed.

Please note, due to the higher lateral resolution in the left image of Fig. 4 and in Fig. 5 D, the Rayleigh range is decreased compared to the corresponding images with the ASM. In consequence, the signal intensity is inhomogeneous. To account that, one can use, e.g., techniques for brightness equalization at the cost of increased noise in the dark regions. Edge preserving noise filters may be used to reduce the amplified noise. For the sake of brevity, the result is not presented here.

## Discussion

We developed a low-cost, easy-to-implement optical add-on for ultra-high-resolution corneal OCT imaging, which is compatible with the commercial SPECTRALIS OCT platform. This prototype achieves a fivefold increased lateral resolution compared to the ASM, albeit a threefold decreased FOV. While the ASM is well suitable for obtaining a quick overview of the cornea, Schlemm’s canal imaging, and anterior chamber angle assessment, the proposed optical add-on, with its increased lateral resolution, provides detailed insight into corneal microstructure, facilitating differentiation between pathological and physiological features.

Comparing in vivo images of the human cornea (cf. Fig. 4), the ASM image is evenly illuminated while the image of the prototype is not. The images taken with the prototype exhibit a curved region with increased brightness where the image appears sharper than in the surrounding areas. This can be explained by the decreased depth of focus (DOF) due to the stronger focusing. At even shorter focal lengths, this effect becomes more pronounced, impeding the direct applicability for microstructural imaging [22]. The observed variation in signal-to-noise ratio between the objective lenses is primarily attributed to the varying fields of view, which lead to reduced laser intensity in the ASM compared to the prototype.

The comparison in Fig. 5 demonstrates that the improved lateral resolution offers a strong benefit for clinical diagnostics. While the ASM image was not suspicious, the higher-resolution image captured with the prototype optical add-on enabled the representation of pathological changes. Supplementary slit lamp images of the same eye can be found in [12]. This is an example of how optimized OCT resolution contributes to better detection and assessment of corneal diseases in medical practice. The prototype provides a valuable enhancement that, owing to its straightforward design, enables rapid and cost-effective optimization in anterior segment examinations.

## Conclusion

This study demonstrates that a compact, low-cost, and technologically simple optical add-on can transform a standard retinal OCT platform into a tool for ultra-high-resolution corneal imaging without major hardware modifications. By increasing the numerical aperture, the prototype achieves about 3 µm lateral resolution - five times higher than a conventional anterior segment module - while preserving compatibility with existing clinical workflows. Although this improvement comes at the cost of a reduced field of view and depth of focus, the gain in structural detail enabled the visualization of epithelial and stromal microfeatures that are otherwise inaccessible to commercial systems. Such detail has direct clinical relevance for early diagnosis, surgical planning, and post-treatment monitoring in a range of corneal conditions, with epithelial basement membrane dystrophy serving here as an illustrative example. The approach offers an accessible and easily implementable pathway to enhance anterior segment OCT performance in both research and routine practice.

## CRediT authorship contribution statement

**Fritz Stange:** . **Sebastian Bohn:** Writing – review & editing, Visualization, Supervision, Methodology, Investigation. **Alois Gottschlich:** Writing – review & editing, Writing – original draft, Investigation. **Tobias Korn:** Writing – review & editing, Supervision, Conceptualization. **Oliver Stachs:** Writing – review & editing, Validation, Supervision, Investigation, Funding acquisition, Conceptualization. **Karsten Sperlich:** Writing – review & editing, Validation, Supervision, Project administration, Methodology, Investigation, Funding acquisition, Conceptualization.

## Declaration of competing interest

The authors declare the following financial interests/personal relationships which may be considered as potential competing interests: The authors declare the following financial interests/personal relationships which may be considered as potential competing interests: The authors declare the following financial interests/personal relationships which may be considered as potential competing interests: Karsten Sperlich: financial support German Research Foundation (DFG grant 469107515).
